# [Retracted] Aberrant expression and mechanism of miR-130b-3p/phosphatase and tensin homolog in nephroblastoma in children

**DOI:** 10.3892/etm.2026.13058

**Published:** 2026-01-05

**Authors:** Yifeng Hu, Jingtie Yan

Exp Ther Med 18: 1021–1028, 2019; DOI: 10.3892/etm.2019.7643

Following the publication of this paper, it was drawn to the Editor’s attention by a concerned reader that flow cytometric (FCM) assay data featured in Fig. 5B were strikingly similar to FCM data which were ultimately published in a number of other papers in different journals that were written by different authors at different research institutes, a few of which had already been submitted for publication prior to the receipt of this article at *Experimental and Therapeutic Medicine.* In addition, the western blot data shown in Fig. 3E were strikingly similar to data which also appeared in a paper written by different authors at different research institutes, albeit these very similar-looking data in that paper were shown in the horizontally reversed orientation.

Owing to the fact that the contentious data in the above article were found to be strikingly similar to data that have been published elsewhere in the scientific literature, the Editor of *Experimental and Therapeutic Medicine* has decided that this paper should be retracted from the Journal. The authors were asked for an explanation to account for these concerns, but the Editorial Office did not receive a reply. The Editor apologizes to the readership for any inconvenience caused.

